# Information and Communication Technology and Organizational Performance During Covid-19 Pandemic: The Role of Organizational Commitment, Growth Mindset, and Entrepreneurial Orientation

**DOI:** 10.3389/fpsyg.2021.752193

**Published:** 2021-09-29

**Authors:** Zhiwen Li, Harold Guy Akouatcha, Umair Akram, Oswin Aganda Anaba

**Affiliations:** ^1^School of Management, Jiangsu University, Zhenjiang, China; ^2^School of Applied Science and Arts, Bolgatanga Technical University, Bolgatanga, Ghana

**Keywords:** ICT adoption, organizational commitment, growth mindset, entrepreneurial orientation, organizational performance

## Abstract

The purpose of this study is to assess how information and communication technology (ICT) adoption influences organizational performance (OP) during the Covid-19 pandemic by highlighting psychometric variables such as employees’ organizational commitment (OC), growth mindset (GM), and entrepreneurial orientation (EO). Based on the complementarity theory, we built a theoretical framework where OC, GM, and EO mediate the influence of ICT on OP and tested hypotheses proposed. Responses of 297 employees from agriculture cooperatives in Côte d’Ivoire were obtained on the basis of questionnaires which composed the data for this study. The empirical analysis affirmed the significant and positive effect of ICT adoption on OP, and the significant mediating effect of OC and GM in the relationship between ICT adoption and OP. However, the role of EO in mediating the influence of ICT adoption on OP is insignificant. This research increases understanding of the underlying process of the relationship between ICT adoption and organizational performance during the Covid-19 pandemic.

## Introduction

In many organizations, technological advances have completely restructured organizations by making their business processes more efficient and fluid than ever. Particularly, information and communication technology (ICT) has become an essential tool to support and sustain the business. This has led companies to invest in ICT as a strategic resource that can directly influence organizational performance (Hermawan and Suharnomo, 2020). Today, ICT has moved from a traditional administrative support orientation to a competitive strategic weapon (Bobb and Harris, 2011; Knight and Radosevich, 2011), because it not only supports existing business operations of organizations, but also enables new business strategies.

Many studies have examined the impact of ICT on organizational performance (Kariuki, 2015; Wu et al., 2015; Shonubi and Akintaro, 2016). Most of these studies have suggested that ICT plays a critical role in improving the quality, quantity, and sharing of information (e.g., Mohout and Fiegenbaum, 2015; Pérez-López et al., 2019), and this enables organizations to allocate their resources to maximize their goals. Laban and Deya (2019) simplify these assertions, stating that organizations use innovation-enhancing ICT to commercialize their products and increase their competitive advantage. This is supported by many previous researches (e.g., Everett, 2003; El-Gohary, 2012; Oliveira et al., 2014) which show that compatibility can help organizations to achieve better performance. Furthermore, other studies highlight that ICT tools allow organizations to leverage formal and informal networks as well as new marketing tactics and, in some circumstances, changes in sales to access markets (Naidoo, 2010).

However, there are still many open questions regarding the hypothetical positive relationship between ICT adoption and organizational performance (Basri et al., 2018), so caution should be exercised when stating that ICT provides organizations with high performance (Jalagat and Al-Habsi, 2017). Some researchers have observed that misalignment of ICT with operations leads to failure to achieve desired outcomes. Yunis et al. (2017) emphasize the need to understand not only how, but also when and why ICT does or does not improve organizational performance. Such disagreements suggest the importance of understanding “how” and “if” ICTs impact organizational performance. In examining how ICT works in organization, Alshubiri et al. (2019) propose to analyze not only the financial return that ICT brings to the company. This view is supported by Lochovsky et al. (1996) who argue that the use of financial measures, in and of itself, does not fully reflect the benefits that ICT brings to the organization. However, Piabuo et al. (2017) advocate ICTs’ integration into human resource management to achieve organizational performance. Their work was based on the effect of ICT usage as a tool for organizational performance through human resources, because ICT can help coordinate human resources, activities, individuals within organizations, and relationships that organizations have at the both intra-organizational and inter-organizational levels.

Overall, the extant literature suggests that the impact of ICT on organizational performance is indirect and that it needs to interfere with other human attributes of an organization to achieve this effect. In this sense, ICTs appear as determinants with significant potential to catalyze other psychometric factors of human resources. This means that ICT capabilities can have a significant impact on firm performance, but only through psychometric attributes of the employees. Several observational studies support this by showing the catalytic and enabling role of ICT (Galve-Górriz and Castel, 2010; Piabuo et al., 2017). However, given that organizational capabilities are many and diverse, research is still needed to clarify and improve the understanding of the enabling role of ICTs on human resources and their impact on outcomes.

This research makes several contributions. First, we provide and test a new explanatory model of the direct and indirect relationships between ICT adoption and the organizational performance through psychometric variables of employees. In this sense, this study contributes to the theoretical understanding of the catalytic and enabling effects of ICTs, which makes us to reinforce the idea that psychological development of employees is essential in the effect of ICTs on the organizational performance. This enables us to not only highlight the effect of ICT in organizations that are often characterized by limited resources, but also emphasize the importance of human attributes catalyzed by ICT adoption which have synergy and significant potential for value creation in organizations.

Second, this study, based on the complementarity theory, incorporates variables such as organizational commitment, growth mindset, and entrepreneurial orientation to understand the enabling capacity of ICT on organizational performance. The complementarity theory provides an entirely different critique by emphasizing that ICTs alone are not enough to revitalize the organizational performance, especially in times of crisis. ICTs need to be complemented by other human attributes such as capabilities, special talent of workers, orientation and proactive spirit of workers, as presented in various studies that link the impact of ICT diffusion on organizational performance to human resources. The human attributes considered in this study include (1) organizational commitment, which is defined as an employee’s level of psychological attachment to his or her organization and, therefore, willingness to strive to achieve organizational goals (Meyer and Allen, 1991); (2) growth mindset, which is defined as an individual’s state of mind that influences outcomes, through a series of socio-cognitive motivators (Dweck, 2006; Boyd, 2014); (3) entrepreneurial orientation, which is defined as the entrepreneurial posture and strategic direction adopted by employees to seize opportunities and is normally manifested by innovative behavior, proactivity and risk-taking propensity, aggressiveness, and competitiveness (Galve-Górriz and Castel, 2010; Gargallo and Galve, 2012). To the best of our knowledge, studies that consider relationships between ICT, OC, GM, EO, and OP are rare.

Third, this study provides a piece of evidence on how ICT adoption works on organizational performance in a very particular context of Covid-19. Despite the existence of studies, little attention has been paid on how the adoption of modern technology affects the organizational performance in times of crisis. Though ICTs have been widely adopted with the development pandemic around the world, the impact of their adoption on the organizational productivity has not received adequate research attention during this period. This research fills the research gap and provides management implications for organizations to adapt their strategies, processes, structure, and culture in order to maximize the contribution of ICT in this pandemic period.

This paper is organized into six sections, beginning with the introduction. “Related Theory, Literature Review and Research Hypotheses” presents the related theory, literature review, and hypothesis development. The methodology of the study is presented in “Materials and Methods.” “Structural Model” presents the results obtained. “Discussion and Conclusion” provides the discussion and the conclusions, and limitations and future research are provided in “Limitations and Future Research.”

## Related Theory, Literature Review, and Research Hypotheses

### Complementarity Theory

It is demonstrated that ICTs reduce transaction and coordination costs, therefore increasing transaction value. They enable businesses to reduce their coordination costs in comparison with their procurement and inventory costs, as well as their coordination costs with suppliers, by improving external communication, reducing inefficiencies caused by a lack of coordination between parties involved, and increasing the speed and reliability of data processing and transfer. Other recent studies on the subject have found that ICT has a beneficial impact on a variety of business performance measures (Bayo-Moriones et al., 2013; Tarutė and Gatautis, 2014; Wachira et al., 2014; Kariuki, 2015; Sabherwal and Jeyaraj, 2015; Mithas and Rust, 2016).

However, the complementarity theory, on which our research is based, proposes that ICT alone is insufficient to provide good productivity, efficiency, and organizational performance (Piabuo et al., 2017). Other human characteristics such as capabilities, specific skills, orientation, and a proactive mindset must be considered, without which organizational effectiveness cannot be achieved (Gargallo and Galve, 2012).

Despite the fact that ICT is widely available, they might be difficult to provide a sustainable competitive advantage on their own. In light of this philosophical divide, recent empirical literature has begun to reconsider the relationship between ICT and a wide range of additional factors. Many scholars have shown that the benefits of using ICT are closely linked to the expression of factors inside the organizational employees who use them (Sarayani et al., 2014). Arvanitis and Loukis (2016) provide empirical evidence of the positive influence of ICT, human capital, and new organizational practices on workplace productivity in Greece and Switzerland. Black and Lynch (2001) investigate the influence of information technology, human resource management practices, and business reorganization on productivity. As a result, ICT will have a significant influence on the organizational performance owing to the adequacy of these human resources (Galve-Górriz and Castel, 2010; Gargallo and Galve, 2012). Following the review of literature, we will focus our attention on the factors are related to human resources, such as organizational commitment, growth mindset, and entrepreneurial orientation.

### Hypotheses Development

#### ICT Adoption and Organizational Performance

ICT’s strategic role is to develop new services and capacity which gives any organization strategic advantages over existing competitive market forces. A company must use its ICTs appropriable and must conform to its internal resources and organizational processes to achieve its long-term success and sustainability (Wu et al., 2015). The choice of ICT provides the compatibility, relative advantage, observability, complexity, and testability for organizations (Everett, 2003).

Compatibility is the dimension that conforms to current values, past experiences, and potential adopters’ needs (Sahin, 2006). If ICTs are compatible with the needs of employees, uncertainty will be reduced, and the rate of adoption will increase. The degree to which an innovation is perceived to be superior to the idea it replaces is referred to as relative advantage. Furthermore, if employees see that technology can add value to their tasks, they will undoubtedly use it (Lee et al., 2019). The successful incorporation of technology into organizations results in the benefits they provide (Shonubi and Akintaro, 2016).

Observability is the result of visible innovation to other adopters. A role model (or peer observation) appears to be a key motivator in the adoption and diffusion of technology by other employees (Everett, 2003). According to research, all of these factors influence the likelihood of employees adopting a new technology in their operations.

Complexity is the degree to which an innovation perceives itself to be difficult to understand and apply, and the extent to which an innovation can be tested on a limited basis is referred to as testability (Everett, 2003). The more times an innovation is tried, the faster it will be adopted.

However, while ICTs have a wide range of applications and benefits for organizational competitiveness, the manner in which these benefits manifest depends on the person–organization–equipment relationship (Sibanda and Ramrathan, 2017). Indeed, it must be acknowledged that some organizations are dissatisfied with the use of these tools as a result of a misalignment of ICT use in these organizations (Silva and Souza Neto, 2014). At this stage, ICT can interfere with organizational performance (Kim and Kim, 2020), which can have a negative impact on costs, sales, and productivity (Wu et al., 2015). Based on this, we believe that ICT, as a strategic tool, has an impact on organizational performance and propose the following hypothesis:

Hypothesis 1(H1): *ICT adoption is positively related to OP*.

#### ICT Adoption and Organizational Commitment

Several studies attest to the possible relationship between communication and commitment (Allen, 1992). The literature on the ICT–commitment relationship has been primarily based on the theory of social exchange, which explains a mechanism of continuous exchanges between various parties. Starting with the theoretical principle of social exchanges, which is based on the concept of reciprocity, the receiver receiving important information from the sender is subject to the full force of the reciprocity law (Emerson, 1976).

A leader who communicates frequently by providing more accurate and timely information improves the work environment and, as a result, increases employee commitment to the organization (Mathieu and Zajac, 1990). Sharing information about the tasks to be completed is still an important prerequisite for organizational commitment. Communication variables can explain more than half of the variance in organizational commitment, and attention and insight, in particular, are communicative predictors of organizational commitment (Hayase, 2009). Furthermore, communication serves as a catalyst for employee attitudes, thereby creating the conditions for commitment (Wiley, 1988; Allen, 1992; Rodwell et al., 1998; Postmes et al., 2001).

This is consistent with the conclusion that the satisfaction of the communication relationship strengthens the individual’s sense of belonging to the organization. When researchers investigated the role of communication, they discovered that openness and relevance of information predicted commitment but not participation in decision-making (Trombetta and Rogers, 1988; Putti et al., 1990). As a result, we propose the following hypothesis:

Hypothesis 2(H2): *ICT adoption is positively related to OC of employees*.

#### ICT Adoption and Growth Mindset

Organizational performance can be defined as the outcome achieved in relation to the objectives, the company’s strategy, and/or the parties’ expectations. Companies need tools and resources to develop their mobility and creativity in order to be successful. Communication channels have been shown to be one of the most important means in business because they play a critical role in achieving a common goal of the organization’s objectives, particularly within a team (Olugbode et al., 2007; Tomal and Jones, 2015).

This adoption of new digital tools or mobile applications results in organizational technological change, which is strongly influenced by an open line of communication between management and employees, a growth mindset, a culture of freedom to choose and innovate, and a shared vision and goal. A strategy like this also contributes to the development of a “culture of growth mindset” which embraces constant change and allows employees to contribute and engage within their organizations.

Hypothesis 3(H3): *ICT adoption is positively linked to GM of employees*.

#### ICT Adoption and Entrepreneurial Orientation

Several studies have shown that ICTs can play an important role in a company’s entrepreneurial orientation (Kietzmann et al., 2013; Sena, 2013). An organization that chooses ICT makes an important decision, often of a strategic nature, allowing it to adjust, integrate, reconfigure, and recreate its internal and external skills in order to gain a competitive advantage in a constantly changing business environment.

Thus, ICTs facilitate interaction with and among stakeholders, resulting in greater agility in specific activities, increased productivity, efficiency, task control, and financial benefits (Jiya, 2019; Pérez-López et al., 2019). Entrepreneurial individuals, for example, associate ICT with many personality traits, including innovation, risk taking, proactivity in the sense of doing what is necessary to bring their ideas to fruition, and responsibility for accepting success or failure (Covin and Slevin, 1989; Morris and Sexton, 1996).

Furthermore, the implementation and creative use of ICT is critical to the success of firms. According to the definition, EO is the process of creating value in conjunction with a unique combination of concepts in order to capitalize on an opportunity (Morris and Sexton, 1996). A possible correlation with strategic positioning was demonstrated by previous studies, implying that entrepreneurship is perceived as a lever of sustainable development for organizations.

So, ICT has the potential to contribute to the development of EO in organization. In this context, the hypothesis is framed as follows.

Hypothesis 4(H4): *ICT adoption is positively linked to EO of employees*.

#### Entrepreneurial Orientation and Organizational Performance

The EO refers to the process of identifying and exploring market opportunities (Venkataraman and Shane, 2000). Several researchers have operationalized the EO through three dimensions: innovation, proactivity, and risk acceptance (Lumpkin and Dess, 1996). Innovation refers to the desire to support and create opportunities for creativity and experimentation in the development of new products. Proactivity is the ability of companies to grow and not just seize market opportunities. The acceptance of risk is reflected in the willingness of top management to allocate a significant part of the company’s resources to new projects or to go into debt in the development of new opportunities. Thus, companies with a strong EO have the ability to manage environmental uncertainties to their advantage to explore opportunities, renew and rejuvenate companies (Covin and Slevin, 1989).

However, companies that do not pay attention to performance indicators and management compromise operating results and their sustainability in the market. Organizations tend to be more seriously affected by changes in the environment, at the level of competitors, suppliers, customers, and other organizations (Wang et al., 2014). Companies need to consider the entrepreneurial posture as an indispensable factor in acquiring skills and, therefore, a competitive advantage in the face of rapid changes in market demands. Thus, based on these researches, we make the following hypothesis:

Hypothesis 5(H5): *EO of employees is positively linked to OP*.

#### Growth Mindset and Organizational Performance

The state of mind was viewed as a required attitude for achieving objectives, which has a significant impact on performance assessments (Heslin et al., 2005; Black and Allen, 2017). A growth mindset supports goal formulation in the first place, goal exploitation in the second, and goal tracking in the third through acquiring a new skill or mastering a task (Dweck, 2006; Yeager and Dweck, 2012). There is a strong link between a growth mindset and self-efficacy (Chunting et al., 2020). As a result, employees with a growth mindset are seen as more resilient in the face of adversity, challenges, and reversals (Yeager and Dweck, 2012). It was believed that individuals’ growth mentality is directly related to their willingness to take risks, face challenges, and work hard (Dweck, 2006). The higher a person’s mental state, the more significance she places on problems and the more time and effort she devotes to improving her condition (Liu et al., 2014). It has been demonstrated, for example, that increasing effort, energy, and tenacity in the execution of learning activities encourages learners to provide their best (Yeager et al., 2019). Persons with a growth mindset try new strategies and ask for help when they need it, in addition to working harder, which is a proven predictor of success (Claro et al., 2016).

As a result, growth mindset of employees can be a substantial factor in promoting organizational performance (Heslin et al., 2006; Boyd, 2014; Asbury et al., 2016).

Hypothesis 6(H6): *GM of employees is positively linked to OP*.

#### Organizational Commitment and Organizational Performance

Organizational commitment remains one of the cornerstones of organizations because of the attitudes and perceptions it generates among the actors of an organization (Folorunso et al., 2014). Existing meta-analyses have indicated that organizational commitment is associated with organizational performance, which is one of the most critical objectives of an organization (Mathieu and Zajac, 1990; Jaramillo et al., 2005).

Employees with highly engaged individuals perceive and consider the objectives, values, and interests of their organizations as their own. Therefore, they make sincere efforts to meet the expectations of the organization by working harder. These efforts are perceived through improved performance, in daily tasks by increasing the quality of employee attitudes and behavior (O’Reilly and Chatman, 1986; Baca-Motes et al., 2013; Grashuis and Cook, 2019). This consolidated commitment can spread to the collective level, to all employees through a social contagion effect, thus forming a “common ground” (Burt, 1987).

If collective commitment results from social interactions within an organization, employees may be inclined to be loyal to the organization, while investing their cognitive, emotional, and physical resources to achieve organizational goals (Burt, 1987; Ostroff, 1992). This collective commitment fosters peer-to-peer support that provides an increased level of cooperative behavior to achieve the organization’s objectives. These behaviors promote quality interactions that are conducive to the achievement of various work-related tasks and cohesion within an organization. Thus, improved attitudes and behaviors would directly contribute to increased organizational efficiency and performance (Andrew, 2017; Ehijiele, 2018).

Hypothesis 7(H7): *OC of employees is positively linked to OP*.

#### The Effect of Organizational Commitment in the ICT-OP Link

As mentioned above, the relationship between ICT and OP has been theoretically and empirically validated in previous work (Tarutė and Gatautis, 2014; Pérez-López et al., 2019). Previous research has shown that ICT adoption can influence performance through organizational commitment (Allen, 1992; Trechter et al., 2002; Sarayani et al., 2014). Since the use of ICT offers advantages by replacing part of human work in an organization on the one hand, and on the other hand, by bringing about greater participation and involvement of employees in new forms of work and in the opening of interpersonal relationships. They are likely to affect positive employee attitudes, such as their commitment to the organization.

If an organization regularly distills information within it, employees will feel more confident, more secure, in order to accomplish their tasks effectively and efficiently, which would increase performance.

Hypothesis 8(H8): *ICT influences OP through OC of employees*.

#### The Effect of the Growth Mindset in the ICT-OP Link

The adoption of ICT in a growth mindset allows employees to improve their information processing and strategic learning skills, allowing them to adapt coordination to changes and environmental dynamics through the resynthesize of resources (Kariuki, 2015; Campbell et al., 2020). It is expected that the effect of ICT on organizational performance relies on employees’ growth mindset.

Hypothesis 9(H9): *ICT influences OP through GM employees*.

#### The Effect of Entrepreneurial Orientation in the ICT-OP Link

ICT has the potential to contribute to the development of entrepreneurial orientation. This perspective gives ICT the role of catalyst that affects perceptions, attitudes, and entrepreneurial behavior in the organization, which is categorized at the micro level (Scott, 1995), and in turn significantly influence various institutional outcomes, such as organizational performance. Thus, we expect the effect of ICT to have a direct impact on organizational performance through the micro-variable of employees’ entrepreneurial orientation. In other words, EO can mediate the ICT-OP link.

Hypothesis 10(H10): *ICT influences OP of through EO of employees*.

**Figure 1:**
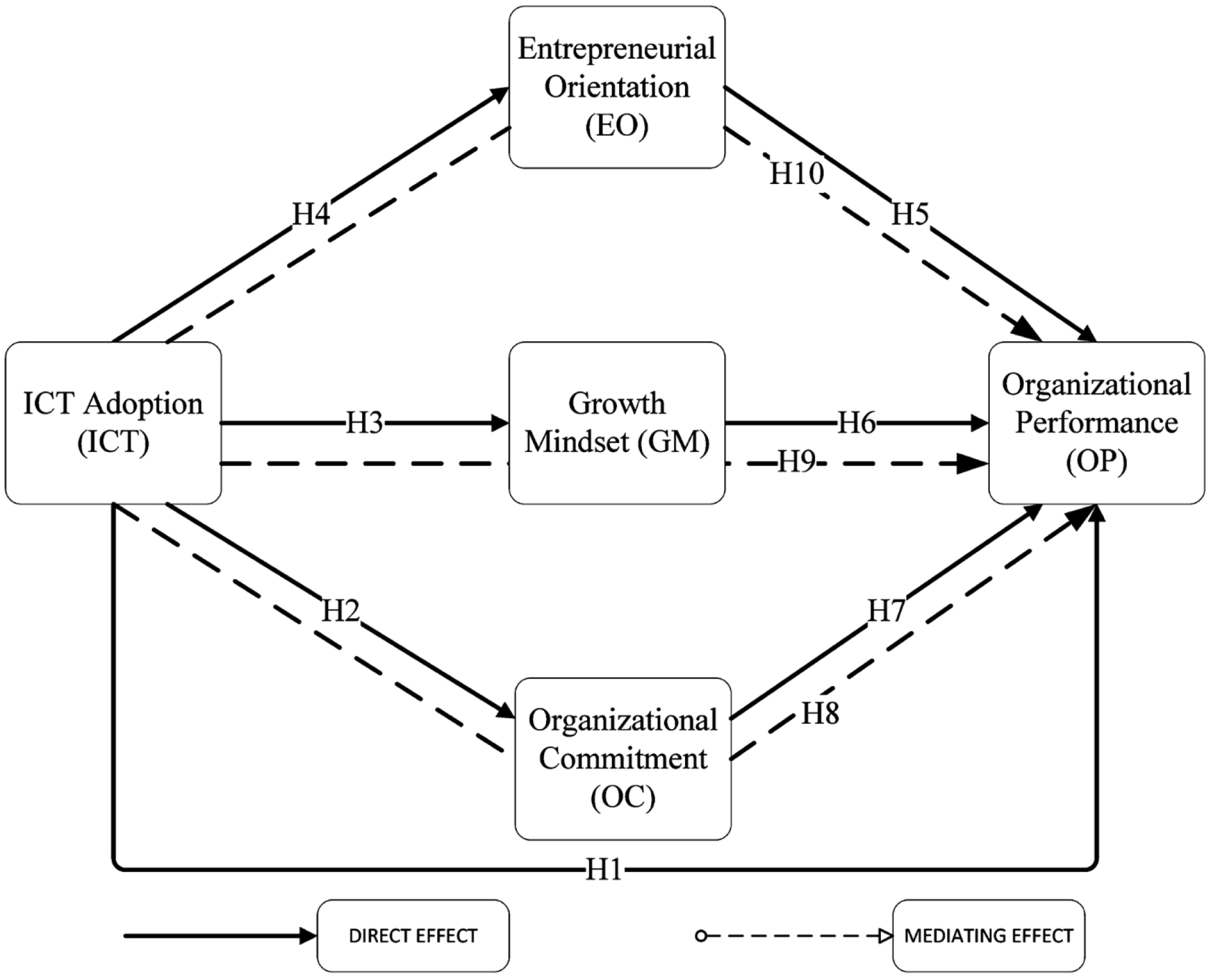
The proposed theoretical framework.

Overall, we propose that impact of ICT on OP through a series of indirect effects of OC, GM, EO (see Figure 1).

## Materials and Methods

### Data Collection

We used an online survey system to collect data from employees who worked in Ivorian agricultural cooperatives. Two reasons explain why we focus on the agricultural cooperatives in Côte d’Ivoire. First, comparing to those in other industries, most agricultural organizations do not develop technological planning strategies in Côte d’Ivoire. This weakness was quickly highlighted by the onset of the pandemic which considerably impacted their functioning. During the pandemic, agricultural organizations initiate more firmly than ever to embrace ICTs and integrate them into their business. This provides us an ideal setting to examine the impact of ICT adoption on organizational performance through employees’ psychological evolution in this transition period. Second, the scale of agricultural organizations in Côte d’Ivoire provides us reliable sample. It is estimated that 37.3% of the Ivorian national GDP comes from agro-industry, according to the Ivorian Ministry of Agriculture. There are around 3,000 professional agricultural organizations, known as cooperatives, scattered across the country. These agricultural professional organizations include Umbrella associations, Coop-CA (Cooperative Society with Board of directors with 15 or 250), agricultural SMES (with 10 to 250 employees), SCOOPS (simplified cooperative with 5 to 15 employees), and VSEs (very small enterprise with less than 10 employees). The cooperatives generate around 100,000 direct jobs, with around 900,000 and 4,000,000 people directly and indirectly linked to these organizations.

The survey targeted respondents from several areas in Côte d’Ivoire (namely Abidjan and Adzopé, Daoukro, Tiassalé) in October 2020. To reduce the harmful effects of sampling bias, we used a method of simple random sampling to select the appropriate quota according to the following four categories: transformation, commercialization, production, and services. After 4weeks investigation, our survey remained online for an additional 15days, allowing respondents to complete it on time. After that, we closed the survey and removed incomplete and missing data from the raw data set. Specifically, of the 378 questionnaires administered, 297 usable questionnaires were obtained, a response rate of 78.57%. The results of the demographic analysis are detailed and presented in Table 1.

**Table 1:** Description of the respondents.

Characteristics of respondents		Frequency (*n*)	Percentage (%)
Gender	Male	201	67.7
	Female	96	32.3
Age	21–29years old	62	20.9
	30–39years old	126	42.4
	40–49years old	61	20.5
	Above 50years old	48	16.2
Marital Status	Single	60	20.2
	Married	177	59.6
	Divorced	39	13.1
	Widower	21	7.1
Education Background	Primary	39	13.1
	High School	182	61.3
	Diploma	38	12.8
	Bachelor’s Degree	21	7.1
	Other	17	5.7
Seniority	1–3years	35	11.8
	4–6years	107	36.0
	7–10years	73	24.6
	Above 10years	82	27.6

### Measures

A self-administered and structured questionnaire was developed, pre-tested and finally administered to respondents while ensuring the anonymity and confidentiality of their responses. A five-point Likert scale has been used to measure variables of research constructs as recommended in previous work (Hamed, 2019). The Likert scale ranged from strongly disagree to strongly agree, coded 1 to 5, respectively. In total, the measurement items for the five multi-item constructs consisted of 19 items that were derived from previous studies and modified to fit the research context. The questionnaire also contained respondent demographics: age, gender, education background, marital status, seniority. Data were analyzed using the Structural Equation Modeling (SEM) with the help of SPSS 21, Amoss21 and Smartpls 3.27.

In order to critically evaluate the influence of ICT on organizational performance of agricultural cooperatives, the following outcomes of SEM-based statistical tests help to justify or nullify the hypothesis of this research. First, before any statistical analysis, all negative statements were recoded. Second, a factor analysis was performed on all ICTs statements and all other categories. For characteristics of ICT adoption, 04 factors (Relative advantage, Compatibility, Complexity, Observability) were produced out of 05. For organizational commitment characteristics, 03 factors (Affective, Normative, Internationalization) were produced out of 05. For characteristics of entrepreneurial orientation, 03 factors (Innovation, Proactivity, Risk-taking) were produced out of 05. For characteristics of growth mindset, 03 factors (to persist or give up after failures, to view the effort as fruitful or unsuccessful, and to learn or avoid comments and criticisms) were produced out of 04. For the characteristics of organizational performance, 06 were produced out of 13. Based on the statements of each factor, the factors were then labeled.

#### Information and Communication Technology as the Independent Variable

ICT adoption has been assessed by employees of agricultural cooperatives using Roger’s DOI scale (Everett, 2003). The scale tends to consider a stakeholder perspective, which consists of items on various modes of adoption for various stakeholders. The scale has four dimensions: (1) Relative advantage (2) Compatibility (3) Complexity (4) Observability. Cronbach’s alpha was 0.924.

#### Organizational Commitment as the 1^st^ Mediator

To measure organizational commitment, we used a combined 3-item model of O’Reilly and Chatman (1986) and Meyer and Allen (1991). Examples of items are: (1) “I really feel that the issues in my organization are mine” (2) “I feel a strong sense of belonging to my organization” and (3) “When I am part of an organization, I feel concerned by the organization itself and not by the bonds of friendship with colleagues.” Cronbach’s *α*=0.959.

#### Growth Mindset as the 2^nd^ Mediator

Growth Mindset is based on a classic preference scale of Dweck (2006) and focuses on the tendencies to persist or give up after failures, to view the effort as fruitful or unsuccessful, and to learn or avoid comments and criticisms. The scale of operationalized measures adapts to the following three items: (1) “When I fail at my job, I see it as an opportunity to learn more” (2) “Comments and criticisms from others motivate me to move forward” (3) “If there is something that I am not very good at, I do not give up until I succeed.” Cronbach’s *α*=0.878.

#### Entrepreneurial Orientation as the 3^rd^ Mediator

Our explanatory variable entrepreneurial orientation symbolized by the entrepreneurial orientation is a composite index which apprehended entrepreneurial intensity within agricultural cooperatives surveyed from a modified version of the index (Shan et al., 2016; Dao, 2018). This index is made up of three dimensions: the propensity to innovation, the propensity to take risks, the propensity to be proactive (Dao, 2018). The questionnaire is reviewed by a research specialist in strategy management and tested on a manager who participates in strategic decisions. Examples of items are: (1) “My organization is embarking on very risky projects” (2) “My organization innovates very often” and (3) “My organization initiates actions to which competitors respond.” Cronbach’s *α*=0.928.

#### Organizational Performance as Dependent Variable

The organizational performance variable was assessed across six items (Cronbach’s *α*=0.936). The examples of items come from previous research and consisted of: (1) “Our organization is more efficient and productive than our competitors” (2) “Our management performance is superior to that of our competitors” (3) “Our financial performance is excellent compared to our competitors” (4) “The return on investment is enormous compared to our competitors” (5) “Value added per employee in our organization is well above the industry average.” and (6) “Productivity of employees is much higher than the industry average” (Kontogeorgos et al., 2018).

### Analytical Strategy

To assess whether measures of ICT adoption included the four-variable structure, we performed confirmatory factor analysis (CFA) to check the validity of the measurement model for our research variables (Marsh et al., 2020; Crocetta et al., 2021). We performed Chi-square difference tests sequentially to compare the quality indices of our five-factor model to other alternative models (i.e., four-factor, three-factor models, and two factors, respectively). This paper used the comparative goodness-of-fit index (CFI), the Tucker–Lewis index (TLI) greater than 0.90 and the approximate root mean square error (RMSEA) less than or equal to 0.06, the (PCLOSE) greater than 0.05 as criteria for evaluating goodness-of-fit indices (Shi et al., 2019). Additionally, we performed a SEM analysis by building a mediation model to test our structural model. We used the maximum likelihood estimator (ML) to perform the SEM. Finally, to assess our mediation hypothesis, we performed a Bootstrap procedure using the 95% bias corrected confidence interval (IC) to assess the mean indirect mediation. If the IC excludes 0, the indirect effects are interpreted as statistically significant at a level of 0.05 (Özdil and Kutlu, 2019).

## Results

### Descriptive Statistics

Table 2 presents descriptive statistics for all variables. As shown in the table, the mean value of ICT was 3.32 with a standard deviation of 0.96. The mean of OC was 3.66 with a standard deviation of 1.10. The mean value of GM was 3.27 with a standard deviation of 1.16, and the mean value of EO was 3.56 with a standard deviation of 1.22. The mean value of OP was 3.47 with a standard deviation of 0.99. The skewness and kurtosis for all constructs in the study show the adequate range of ±2, which agrees with the symmetry of the sample distribution (Andrews et al., 2020).

**Table 2:** Descriptive statistics.

	*N*	Min	Max	Sum	Mean		*SD*	Skewness		Kurtosis	
	Stat	Stat	Stat	Stat	Stat	Std. Error	Stat	Stat	Std. Error	Stat	Std. Error
ICT	297	1.00	5.00	986.25	3.32	.056	0.96	−0.390	0.141	−0.537	0.282
OC	297	1.00	5.00	1087.17	3.66	.064	1.10	−0.621	0.141	−0.527	0.282
GM	297	1.00	5.00	972.33	3.27	.067	1.16	−0.367	0.141	−0.744	0.282
EO	297	1.00	5.00	1058.43	3.56	.071	1.22	−0.608	0.141	−0.816	0.282
OP	297	1.00	5.00	1029.14	3.47	.058	0.99	−0.474	0.141	−0.822	0.282

### Structural Model

#### Confirmatory Factor Analysis of Variables

This research employs five major constructs (ICT, OC, GM, EO, and OP) as major constructs. The study first examines the different reliability of the elements for the measurement model. Initially, the associations between the variables were evaluated by a Pearson correlation analysis (Anderson and Gerbing, 1988). Moreover, CFA has helped to understand that the measurements of the constructor are consistent (Idris et al., 2010; Marsh et al., 2020). The analytical result was used to test whether the data met the hypothetical measurement model. To do this, we conducted a CFA for the 19 elements to verify the quality of the fit of the measurement model of the five variables and we also built three mediation models between ICT adoption and performance. In the conceptual structure, OC, GM, and EO mediated the ICT-OP link and the statistical results based on the model fit indices showed that all values of the CFA indicators (i.e., CMIN/DF, IFI, GFI, CFI, and RMSEA) are within their threshold of excellence. As shown by the observed value of CMIN/DF is 1, 387, which is less than 3, the TLI value is 0.988 (greater than 0.90), the CFI value is 0.991 (greater than 0.90), the NFI value is 0.968 (greater than 0.90), the GFI value is 0.940 (greater than 0.80) and RMSEA 0.036 (greater than 0.08), the Pclose value is 0.973 (greater than 5).

**Figure 2:**
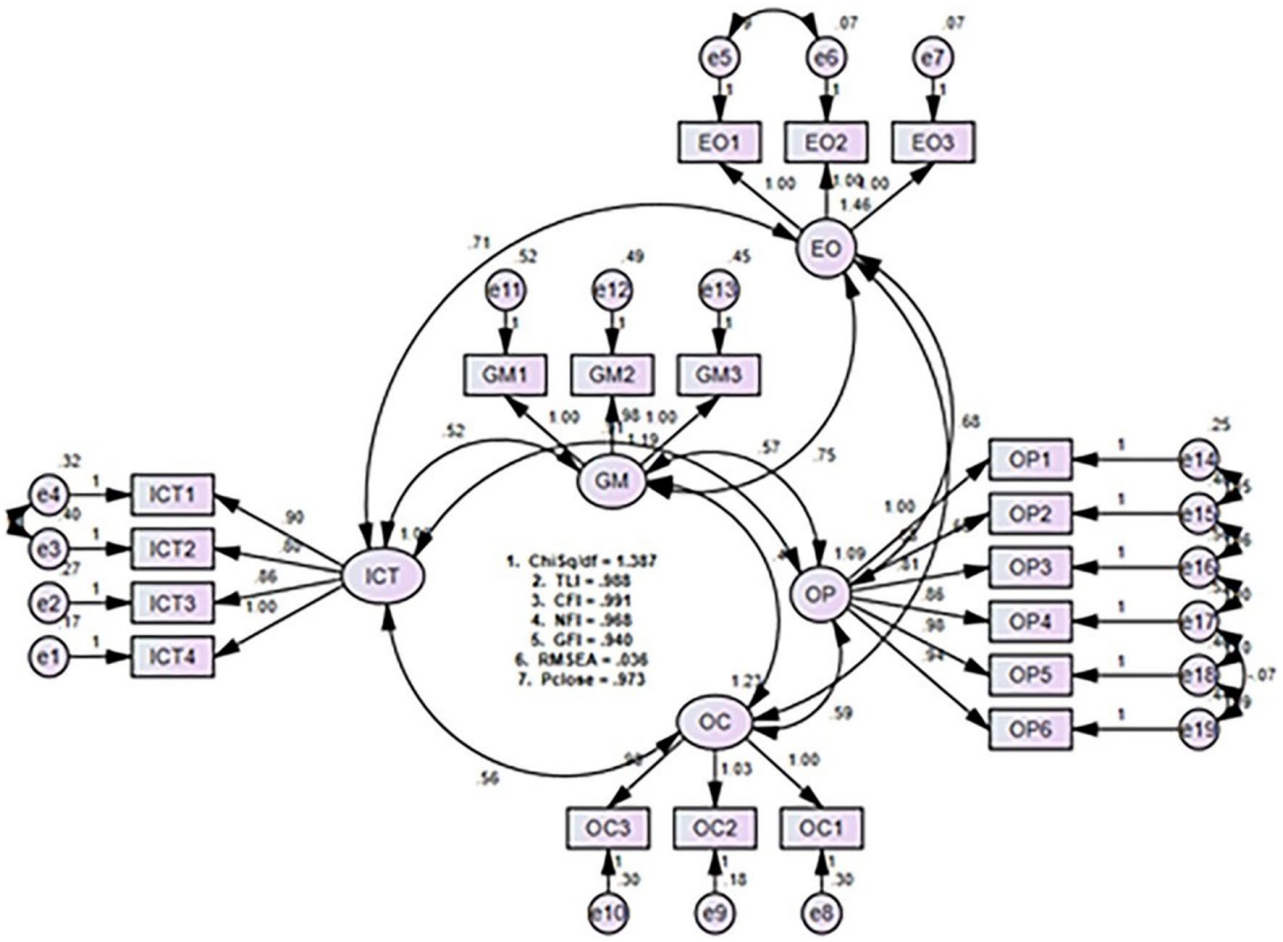
Confirmatory factor analysis.

In addition, a multi-collinearity bias was performed between different variables. We also looked at variance inflation factors (VIF) and tolerances. For ICT, OC, GM, EO, the results are 1.56, 1.38, 1.46, 1.82, respectively, and the tolerance values for these variables are 0.72, 0.51, 0.42, and 0.36, respectively. The VIF scores are below 10, and the tolerance scores are above 0.2. We suggest that ICT, OC, GM, EO are free from the multi-collinearity problem. This means that all variables are effectively and completely uploaded to the CFA model, as shown in the following Figure 2.

#### Results of the Mediation Analysis

We performed SEM analyses and a Chi-square difference test between the full mediation model and the partials mediations models. Our full mediation model is excellent (CMIN=185.852; *df*=134; CMIN/DF=1.387; CFI=0.991; TLI=0.988; NFI=0.986; GFI=0.940; RMSEA=0.36; Pclose=973). The partial mediations models include three indirect paths that connect ICT, OC, GM, EO, and OP. The first partial mediation model (ICT-OC-OP) fits perfectly (CMIN=131.146; *df*=90; CMIN/DF=1.457; CFI=0.991; TLI=0.989; RMSEA=0.39; PCLOSE=890). The second model of mediation involves an indirect path ICT-GM-OP. The partial mediation model also fits perfectly (CMIN=93.615; *df*=54; CMIN=1.734; CFI=0.991; TLI=0.986; RMSEA=0.050; PCLOSE=0.487). The third model of mediation involves an indirect path ICT-EO-OP. The partial mediation model also fits perfectly (CMIN=66.930; *df*=27; CMIN=2.479; CFI=0.984; TLI=0.986; RMSEA=0.071; PCLOSE=0.53). The result of our Chi-square difference test between models demonstrated that the full mediating model was significant and remains the best. Therefore, we suggest that the three variables have an adequate level of discriminant validity.

#### Test of Hypotheses

Table 3 analyzes the construction assumptions, including their beta, mean, standard deviation, *t*, and *p* values.

**Table 3:** Path coefficients and hypothesis tests.

Hypotheses	Relationships	Beta	Mean	*SD*	*t*-value	*p*-value	Result
H1	ICT ->OP	0.667	0.666	0.049	13.669	0.000	Supported
H2	ICT ->OC	0.453	0.454	0.048	9.425	0.000	Supported
H3	ICT ->GM	0.424	0.425	0.046	9.175	0.000	Supported
H4	ICT ->EO	0.542	0.542	0.039	13.890	0.000	Supported
H5	EO ->OP	0.032	0.032	0.048	0.677	0.498	Non-supported
H6	GM ->OP	0.113	0.117	0.049	2.299	0.022	Supported
H7	OC ->OP	0.121	0.121	0.054	2.232	0.026	Supported
** Standardized direct, indirect, and total effects of the hypothesized model**							
H8	ICT ->OC ->OP	0.055	0.055	0.025	2.226	0.026	Supported
H9	ICT ->GM ->OP	0.048	0.050	0.022	2.198	0.028	Supported
H10	ICT ->EO ->OP	0.017	0.018	0.026	0.666	0.506	Non-supported

According to the study’s findings, the reliability and validity of the scales measured for OP are 0.855, 0.897, 0.902, 0.975, and 0.911, respectively. The path coefficients suggested by the model provide empirical support for the 10 hypotheses tested in this study because this technique can be used to test multiple levels of a theoretical framework. Analyses show that ICTs have a positive impact on the organizational performance of agricultural cooperatives.

The results for hypothesis 1 (H1) show a beta coefficient of 0.667, a standard error of 0.049, a *t*-value of 13.669>2, and a *p*-value is 0.000<0.01. Similar results have been discovered to demonstrate that the adoption of ICT plays a critical role in organizational performance, providing cooperatives with a one-sided advantage in their daily tasks (e.g., Bayo-Moriones et al., 2013).

In the test of hypothesis 2 (H2), the beta coefficient is 0.45, the standard error is 0.048, the value *t*=9.425>2 and the value *p*=0.000<0.01. So H2 is supported. This finding is consistent with a recent study which contends that ICT has a positive impact on employee commitment if and only if it is used effectively (e.g., Sarayani et al., 2014; Naser and Sajad, 2016). This entails centralizing information and effectively disseminating information to the appropriate person, at the appropriate location, and at the appropriate time.

According to the findings of this study, ICT has a positive impact on the GM of employees, which supports hypothesis 3 (H3). The results show that the beta coefficient is 0.42, the standard error is 0.046, the *t*-value is 9.175>2, and the *p*-value is 0.000<0.05.

Hypothesis 4 (H4) is justified because the beta coefficient is 0.54, the standard error is 0.039, the *t* value is 13.890>2, and the *p* value is 0.0000<0.01. Similar results were found by the previous study which added that technology sets the outer limits of what can be imagined in terms of new ways of optimizing operations and developing new products and services.

As for hypothesis 5 (H5), the results of the study show the beta coefficient 0.032, standard error 0.048, *t*-value 0.6772. *p*-value is 0.498>0.05 that is recognized as an index quantifying the strength of the evidence against the null hypothesis. So, the hypothesis is not confirmed based on the results.

The results of the study show that a significant impact of GM on OP with the beta coefficient 0.113, standard error 0.049, *t*-value 2.299>2 and *p*-value is 0.022<0.05. Therefore, hypothesis 6 (H6) is supported, and the result echoes previous studies examining the effect of mindset on organization performance (Zingoni and Corey, 2017).

For hypothesis 7 (H7), the results are significant and confirmed by the values of the positive beta coefficient 0.121, standard error 0.054, *t*-value 2.232>2, and *p*-value is 0.026<0.05. These findings are consistent with those of previous studies.

This study used the Smartpls 3.27 software’s bootstrap method to compare the three mediating effects mentioned above (the size of one was set at 5,000 cases to illuminate the path coefficients and their significance, and the interval confidence level was set at 95 percent; Jung et al., 2019). Mediation being described as process through which the independent variable is likely to influence the dependent variable (Baron and Kenny, 1986), we can see through the full representation of structural model evaluations, as well as population mediation statistics, in Figure 2, and Table 3 that the independent variable is responsible for triggering the action of these 3 mediators or their intensity, which itself influences the response (dependent variable). Precisely, it entails showing the relevance of indirect effects *via* mediating factors using a model of analysis execution.

If the indirect effects are not statistically significant, we conclude that no mediating effect exists (Agler and De Boeck, 2017). We used a test procedure to test the hypothesis of mediating effects (Preacher and Hayes, 2004). The mediating effect assumes that the indirect effect is significant and that the confidence interval does not contain zero. The mediating effects of GM and OC assume a positive and meaningful relationship with ICT and PO integration. Table 2 shows that OC (*p*=0.026) and GM (*p*=0.028) both positively and significantly mediate ICT–OP link. As a result, H8 and H9 are all supported, which is consistent with previous studies (e.g., Glaeser, 2016; Nagy et al., 2016).

The results of the study of ICT on OP by the mediation of EO are not significant and are invalidated by the values of the positive coefficient 0.017, the standard deviation 0.026, the *t*-value 0.6662. Furthermore, *p*-value (0.506) is greater than the threshold of 0.05, which shows insignificance. This indicates strong support for the null hypothesis. As a result, H10 is not supported.

## Discussion and Conclusion

The purpose of this study was to shed light on the inconsistent findings of previous studies on the ICT-performance link by considering the supplement of human resource attributes, such as organizational commitment, growth mindset, and entrepreneurship orientation. These variables are known to function as fundamental components of an organization, as they are closely associated with a variety of organizational outcomes (Meyer and Allen, 1991; Caniëls et al., 2018). We proposed that the high level of organizational commitment, growth mindset, and entrepreneurial orientation catalyzed by ICT can improve organizational performance in a crisis context.

Using SEM to conduct an analysis of the mediation model, this study provides empirical evidence that ICT adoption has positive impact on OP of agricultural cooperatives amid pandemic. Based on the findings, we discovered that ICT adoption contributes to OP with the complement of appropriate human resource attributes. Specifically, OC and GM serve as intermediation factors in the relationship between the ICT and the OP, but EO neither has impact on OP nor mediates the ICT-OP relationship. This could be due to various factors specific to agricultural cooperatives and is consistent with the results of previous research which showed that companies with a stronger EO posture often have poorer results (Wales et al., 2011).

Our study differs from previous research in that it combines mediating factors that explain why and when ICT can improve OP. The sections that follow discuss the study’s theoretical and practical implications.

### Theoretical Implications

This paper has the potential to make a significant contribution to the ICT literature for two reasons. First, the study combines macro- and micro-approaches to examine the impact of ICT adoption on organizational performance. The macro-approach emphasizes external factors (e.g., ICT and OP), while the micro-approach highlights internal factors (e.g., human attributes) to explain how ICT influences organizational performance both directly and indirectly. The results obtained by coupling the variables OC, GM, and EO enrich the literature on the value of ICT. Several related works have focused on the relationship between ICT and external stakeholders, with little attention paid to the micro-level aspect, more specifically internal stakeholders, which is the focus of this study.

Second, this study specifically demonstrated that ICT adoption, acting as a critical catalyst, has a significant impact on the behavioral and positive cognitive processes of the employees with regard to their organization. Specifically, ICT adoption leads to a positive growth mindset and organizational commitment, which ultimately increases the organizational performance. It shows that complementarities between ICT adoption and human resources have a positive influence on the growth of businesses. As a result, this study demonstrates the application of the complementarity theory in organizations.

### Practical Implications

The current study has practical and meaningful implications for organization leaders. First, leaders and senior management teams that intend to integrate ICT into their operations during the pandemic must view their employees as important agents who can improve performance. Most importantly, leaders should recognize that employees’ reactions in the form of various perceptions, attitudes, and behaviors toward their organization play a critical role in creating organizational performance. As a result, leaders should attempt to efficiently communicate and interact with their employees *via* ICT. Furthermore, they must provide adequate rewards for employees in order to maximize active participation through them. By communicating with ICT on a regular basis, the leadership team can foster an ICT-driven culture within the organization.

Second, we propose that senior management teams pay more than special attention to their employees’ behavioral indicators, which remain critical to the performance of their organization. Given the importance of the organization’s communication processes in influencing the effects of performance both directly and indirectly, it is critical for organizations to maintain and improve employee perceptions and attitudes. Based on our findings, we propose that senior leadership teams must understand and accept that the level of commitment and a common mental pattern, such as a employees’ growth mindset, serve as a practical indicator of whether ICT can have a positive impact on performance.

## Limitations and Future Research

The following research limitations should be solved in order to favorably contribute to future studies. First, the way employees adopt ICT might be influenced by cultural variations between organizations in poor and industrialized countries. Western cultures are more likely to emphasize the relevance of ICTs in their environments; therefore, their employees may be more receptive to using them for assignments. Because we collected data from Ivorian agricultural cooperative employees, careful interpretation of the results is required when they are applied in the context of different cultural environments (Arnold et al., 2007). Despite the fact that ICT is universal, employees of Ivorian may have reacted differently to ICT use than employees of Western, Eastern Asian. As a result, other studies on the ICT–OP link should account for these cultural differences.

Second, we were unable to include objective indices for measuring ICT. Although previous ICT adoption studies have suggested that subjective measures, such as employee perceptions, may more accurately reflect actual phenomena of performance than objective measures of ICT adoption, we recommend that future work includes both subjective and objective measures of ICT adoption in the research model (Saengchaia et al., 2019).

Third, this paper did not adequately address the potential endogenous problem and its solutions. Endogenous issues are caused by a variety of unobservable factors. For example, ICT, OC, GM, and EO are likely to be heavily influenced by unobservable factors such as skills, business age, business ownership, culture, reputation, and visibility (Ugwu and Ugwu, 2017). Future research should investigate and adequately address this issue.

Fourth, this paper only looked at the roles of OC, GM, and EO as mediators in explaining the ICT–OP nexus. However, it is important to note that a variety of important micro-level variables (for example, motivation, corporate social responsibility, collaboration, trust, participation, and psychological well-being) are likely to act as intermediators to translate the positive influence of ICT on organizational performance. This issue should be addressed in greater depth in future studies.

Fifth, we were unable to fully investigate the significant impact of organizational cultures on employee perceptions of ICT adoption. Organizational cultures such as effectiveness, customer orientation, control, approachability, and management philosophy can all have a significant impact on the level of ICT adoption and employee perceptions of ICT. As a result, future research should investigate the role of organizational cultures in explaining the impact of ICT adoption.

## Data Availability Statement

The raw data supporting the conclusions of this article will be made available by the authors, without undue reservation.

## Author Contributions

Conceptualization and software were contributed by ZL. Data curation and formal analysis were contributed by GA. Methodology was contributed by UA. Writing—original draft was contributed by ZL and GA. Writing—review and editing was contributed by UA and OA. All authors contributed to the article and approved the submitted version.

## Conflict of Interest

The authors declare that the research was conducted in the absence of any commercial or financial relationships that could be construed as a potential conflict of interest.

## Publisher’s Note

All claims expressed in this article are solely those of the authors and do not necessarily represent those of their affiliated organizations, or those of the publisher, the editors and the reviewers. Any product that may be evaluated in this article, or claim that may be made by its manufacturer, is not guaranteed or endorsed by the publisher.
